# Diet and the gut microbiome in patients with Parkinson’s disease

**DOI:** 10.1038/s41531-024-00681-7

**Published:** 2024-04-22

**Authors:** Dayoon Kwon, Keren Zhang, Kimberly C. Paul, Aline D. Folle, Irish Del Rosario, Jonathan P. Jacobs, Adrienne M. Keener, Jeff M. Bronstein, Beate Ritz

**Affiliations:** 1 Department of Epidemiology, UCLA Fielding School of Public Health, Los Angele, CA USA; 2 Department of Neurology, UCLA David Geffen School of Medicine, Los Angeles, CA USA; 3 The Vatche and Tamar Manoukian Division of Digestive Diseases, Department of Medicine, UCLA David Geffen School of Medicine, Los Angeles, CA USA

**Keywords:** Parkinson's disease, Epidemiology

## Abstract

It has been suggested that gut microbiota influence Parkinson’s disease (PD) via the gut–brain axis. Here, we examine associations between diet and gut microbiome composition and its predicted functional pathways in patients with PD. We assessed gut microbiota in fecal samples from 85 PD patients in central California using 16S rRNA gene sequencing. Diet quality was assessed by calculating the Healthy Eating Index 2015 (HEI-2015) based on the Diet History Questionnaire II. We examined associations of diet quality, fiber, and added sugar intake with microbial diversity, composition, taxon abundance, and predicted metagenomic profiles, adjusting for age, sex, race/ethnicity, and sequencing platform. Higher HEI scores and fiber intake were associated with an increase in putative anti-inflammatory butyrate-producing bacteria, such as the genera *Butyricicoccus* and *Coprococcus 1*. Conversely, higher added sugar intake was associated with an increase in putative pro-inflammatory bacteria, such as the genera *Klebsiella*. Predictive metagenomics suggested that bacterial genes involved in the biosynthesis of lipopolysaccharide decreased with higher HEI scores, whereas a simultaneous decrease in genes involved in taurine degradation indicates less neuroinflammation. We found that a healthy diet, fiber, and added sugar intake affect the gut microbiome composition and its predicted metagenomic function in PD patients. This suggests that a healthy diet may support gut microbiome that has a positive influence on PD risk and progression.

## Introduction

Parkinson’s disease (PD) is the second most common age-related neurodegenerative disease, with an increasing prevalence and incidence^1^. PD patients not only suffer from progressive motor impairment but also from extensive non-motor symptoms, including gastrointestinal (GI) dysfunction, constipation, cognitive impairment, and depression^2^. These non-motor symptoms can occur decades before clinical diagnosis during its prodromal stages. While there are treatments that minimize motor impairments, little advancement has been made in efforts to halt disease progression before the onset of motor symptoms.

Growing evidence indicates that in PD patients, diets are affected by or may be affecting the disease. Better diet quality, as measured by the Alternative Healthy Eating Index (AHEI) and the alternate Mediterranean Diet Score (aMED), has previously been associated with a lower risk of incident PD^3,4^ and prodromal symptoms^5^. Our previous study found that PD patients had a lower quality of diet, assessed by Healthy Eating Index (HEI)-2015, AHEI, and aMED, compared to their household and community controls. This difference was primarily due to lower dietary fiber and higher added sugar intake^6^. We also observed lower HEI scores in PD patients with chronic constipation and hyposmia. Additionally, those with cognitive impairment consumed even less fiber. Furthermore, PD patients diagnosed at a younger age or with a longer disease duration tended to consume more added sugar.

Recent research highlights a connection between the gut and brain in PD, with the gut microbiome potentially playing a role in disease onset. As we age, the balance of bacterial communities in the gut changes, resulting in a decrease in beneficial bacteria and an increase in harmful bacteria. Such dysbiosis, associated with age or induced by a poor-quality diet, can lead to inadequate production of essential nutrients and a rise in toxins that cause inflammation, including neuroinflammation and neurodegeneration^7,8^. Compared to healthy controls, PD patients exhibit gut microbiome characterized by a decrease in putative short-chain fatty acid (SCFA)-producing bacteria, such as genera *Butyricicoccus*^9,10^ and *Coprococcus*^11–13^ as well as an increase in putative pro-inflammatory bacteria, such as the genera *Akkermansia*^12,14–17^.

While the importance of dietary habits in shaping the gut microbiome is undisputed, its impact on the development and progression of PD is just starting to be better understood, with specific nutrients such as low fiber and high added sugar intake being identified as potential risk factors^18,19^. Improving gut health through diet could reduce GI-related symptoms and enhance the quality of life in PD, and potentially even show avenues to slow disease progression^20^. Therefore, it is necessary to better understand the influence of diet on the gut microbiome in PD. In this study, we examine the associations of diet (diet quality, fiber intake, and added sugar) with gut microbiome diversity, composition, abundance, and its predicted metagenome in PD patients, with the hope of providing a better understanding of how lifestyle, and especially dietary interventions, may become useful tools in the prevention and/or treatment of PD.

## Results

### Analytic cohort characteristics

Mean age of PD patients at diagnosis in our study was 62 years (range: 41–84), with a mean age of 74 years at the time of fecal sample collection and a PD duration of 9.7 years (range: 2–20; Table 1). Most participants were men (67%) of European ancestry (81%), overweight (mean BMI of 27.1 kg/m^2^), well educated (mean 15.4 years of education), and never smokers. Constipation was found to be higher among PD patients in the lowest HEI score tertile, with 72% of patients experiencing constipation compared to 50% in the highest HEI score tertile.

**Table 1:** Characteristics of PD patients by HEI score tertile

		HEI-2015	
	Low	Intermediate	High
	(*n* = 29)	(*n* = 28)	(*n* = 28)
Age (years)			
Mean (SD)	75.6 (7.88)	73.4 (8.55)	72.5 (9.39)
Range	54–88	51–86	56–90
Sex			
Male	19 (65.5%)	20 (71.4%)	18 (64.3%)
Female	10 (34.5%)	8 (28.6%)	10 (35.7%)
Race/Ethnicity			
White non-Hispanic	24 (82.8%)	22 (78.6%)	23 (82.1%)
Non-White or Hispanic	5 (17.2%)	6 (21.4%)	5 (17.9%)
Platform			
HiSeq	26 (89.7%)	21 (75.0%)	20 (71.4%)
MiSeq	3 (10.3%)	7 (25.0%)	8 (28.6%)
BMI (kg/m^2^)			
Mean (SD)	27.1 (5.56)	28.5 (5.83)	25.8 (5.11)
Range	16.2–39.7	14–41.3	14.8–38.3
Education (years)			
Mean (SD)	14.9 (2.50)	14.5 (3.64)	16.8 (4.77)
Range	12–22	4–22	4–27
Smoking status			
Never	17 (58.6%)	19 (67.9%)	17 (60.7%)
Ever	12 (41.4%)	9 (32.1%)	11 (39.3%)
Alcohol intake (g/d)			
Mean (SD)	2.59 (6.25)	9.24 (17.7)	3.95 (7.47)
Range	0–26.4	0–76.8	0–28.3
Caffeine intake (mg/d)			
Mean (SD)	147 (184)	195 (308)	203 (252)
Range	2.67–611	2.96–1430	0.52–1020
Energy intake (kcal/d)			
Mean (SD)	1940 (914)	2080 (866)	1850 (811)
Range	627–4000	731–4430	653–4050
Age at PD diagnosis (years)			
Mean (SD)	64.3 (9.04)	64.7 (8.46)	62.4 (11.1)
Range	50–84	42–75	41–81
PD duration (years)			
Mean (SD)	10.2 (5.28)	9.14 (3.78)	9.79 (4.84)
Range	4–20	3–20	2–20
Constipation			
No	8 (27.6%)	13 (46.4%)	14 (50.0%)
Yes	21 (72.4%)	15 (53.6%)	14 (50.0%)
MDS-UPDRS IA			
Mean (SD)	3.78 (3.11)	3.77 (2.62)	3.38 (2.98)
Range	0–10	0–11	0–11
MDS-UPDRS IB			
Mean (SD)	11.6 (4.50)	9.33 (4.95)	9.47 (5.20)
Range	4–22	2–24	1–21
MDS-UPDRS II			
Mean (SD)	17.5 (9.09)	16.4 (7.80)	14.8 (8.57)
Range	1–41	3–40	2–40
MDS-UPDRS III			
Mean (SD)	28.3 (12.2)	28.3 (10.7)	24.5 (13.4)
Range	11–58	8–51	3–58.2

*PD* Parkinson’s disease, *HEI* Healthy Eating Index, *SD* standard deviation, *BMI* body mass index, *MDS-UPDRS* Movement Disorder Society-Unified Parkinson’s Disease Rating Scale.

### Diet quality

**Fig. 1:**
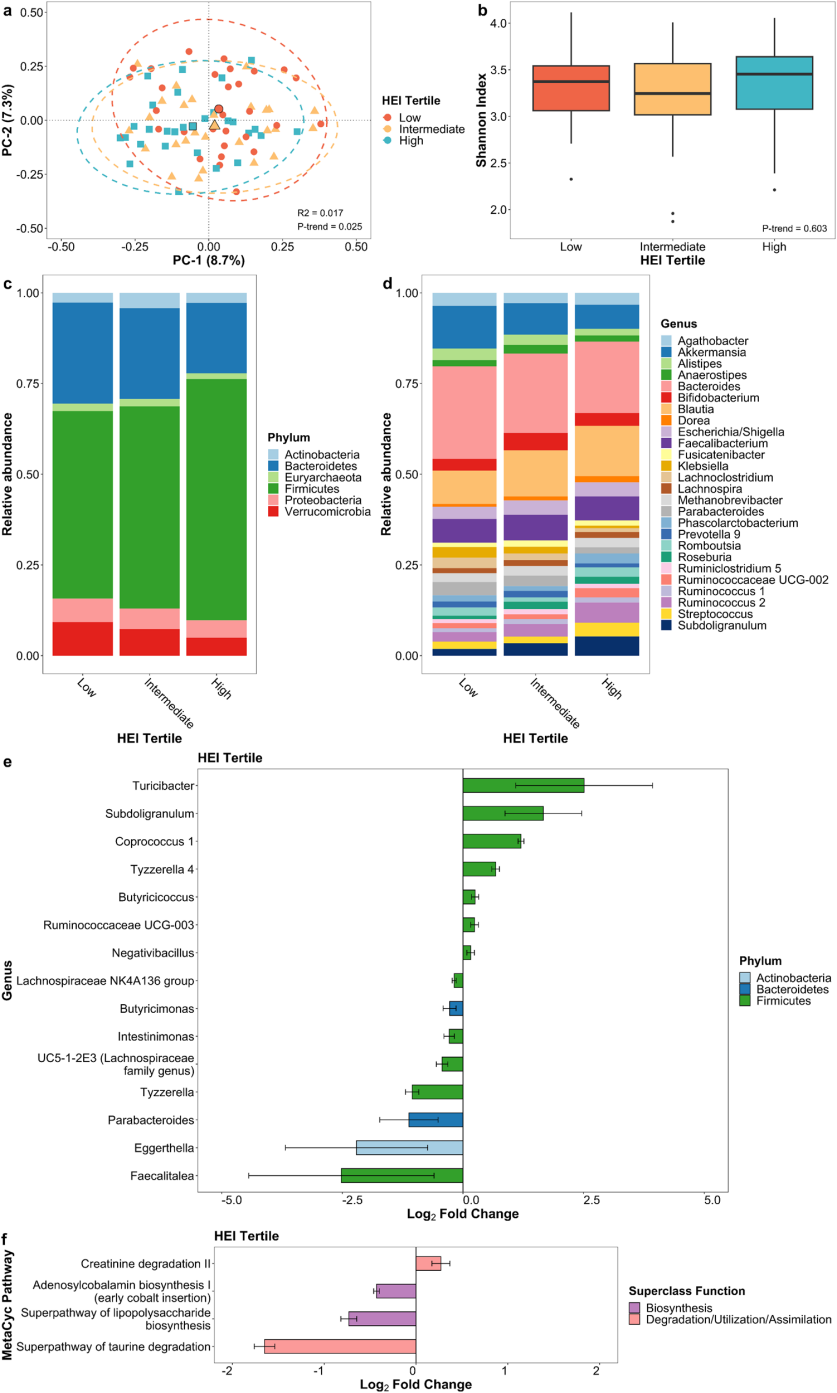
Gut microbiome of PD patients varies by HEI score. **a** Principal coordinate analysis plot of the microbiome by HEI tertile. **b** Box plot of microbial diversity using Shannon index by HEI tertile. Taxonomic summary plot of relative abundance at **c** phylum and **d** genus level (≥1% relative abundance) by HEI tertile. **e** Differential abundance of microbial taxa at genus level and **f** functional pathways from predicted metagenome by HEI score after adjusting for age, sex, race, and sequencing platform. Taxa and pathways in (**e**) and (**f**) are associated with categorical HEI tertile at *q*-trend <0.05 and continuous HEI score at *q*-value < 0.05. Error bars represent 95% confidence intervals.

The HEI score was associated with the beta diversity (*p*-trend = 0.025; Fig. 1a) but not alpha diversity (Fig. 1b) in multivariable-adjusted linear regression models. The first and second principal coordinate axes were negatively associated with increasing HEI score tertiles, indicating significant differences in microbial communities among PD patients across HEI tertiles. To further define compositional differences across HEI tertiles, the crude relative abundance at the phylum and genus levels was computed. When we used multivariable-adjusted negative binomial generalized linear models to examine associations of the HEI with microbial taxa abundance at the genera level, a better diet according to the HEI increases the bacteria *Butyricicoccus* (*q*-trend < 0.001), *Coprococcus* 1 (*q*-trend < 0.001), *Negativibacillus* (*q*-trend < 0.001), and *Ruminococcaceae UCG-003 (q*-trend < 0.001) and decreases *Parabacteroides* (*q*-trend = 0.002) and *Eggerthella* (*q*-trend = 0.016; Fig. 1e; Supplementary Data 1). Exploring microbiota function based on predicted metagenomes, we found differences in predicted metagenomic profiles with increasing HEI scores (*p*-trend = 0.030; Supplementary Table 1) but not in bacterial gene richness (i.e., the number of predicted genes). Differential abundance testing linked four pathways to the HEI, including pathways related to biosynthesis and degradation. Pathways enriched in patients with a higher HEI were related to creatinine degradation II (*q*-trend < 0.001), while pathways less abundant in patients with a higher HEI were associated with adenosylcobalamin biosynthesis I (*q*-trend < 0.001), superpathway of lipopolysaccharide biosynthesis (*q*-trend < 0.001), and superpathway of taurine degradation (*q*-trend < 0.001; Fig. 1f; Supplementary Data 2).

### Dietary fiber intake

**Fig. 2:**
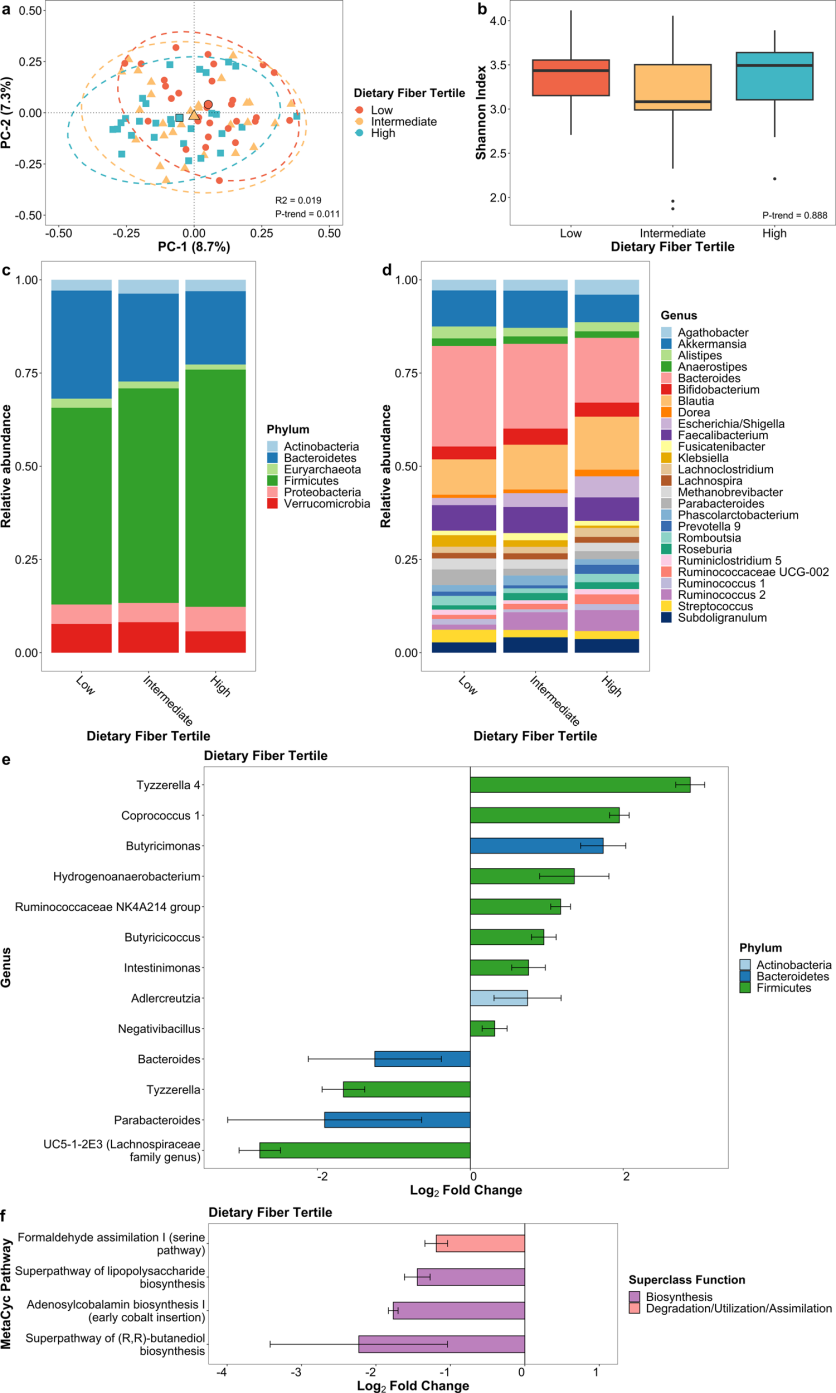
Gut microbiome of PD patients varies by dietary fiber intake. **a** Principal coordinate analysis plot of the microbiome by dietary fiber intake tertile. **b** Box plot of microbial diversity by Shannon index by dietary fiber intake tertile. Taxonomic summary plot of relative abundance at **c** phylum and **d** genus level (≥1% relative abundance) by dietary fiber intake tertile. **e** Differential abundance of microbial taxa at genus level and **f** functional pathways from predicted metagenome by dietary fiber intake after adjusting for age, sex, race, and sequencing platform. Taxa and pathways in (**e**) and (**f**) are associated with categorical dietary fiber intake tertile at *q*-trend <0.05 and continuous dietary fiber intake at *q*-value < 0.05. Error bars represent 95% confidence intervals.

Dietary fiber intake was associated with microbial composition (*p*-trend = 0.011; Fig. 2a) but not alpha diversity (Fig. 2b). As fiber intake tertiles increased, a negative association was observed with the first and second principal coordinate axes. At the genus level, increased *Butyricicoccus* (*q*-trend < 0.001), *Coprococcus* 1 (*q*-trend < 0.001), *Hydrogenoanaerobacterium* (*q*-trend < 0.001), *Negativibacillus* (*q*-trend = 0.001), *Ruminococcaceae NK4A214 group* (*q*-trend <0.001), along with decreased *Parabacteroides* (*q*-trend = 0.018) and *Bacteroides* (*q*-trend = 0.026; Fig. 2e; Supplementary Data 1), were associated with fiber intake. There was a marginal difference in the predicted metagenomic profile (*p*-trend = 0.091; Supplementary Table 1) but not in bacterial gene richness. Four pathways differed in abundance: formaldehyde assimilation I (*q*-trend < 0.001), superpathway of lipopolysaccharide biosynthesis (*q*-trend <0.001), adenosylcobalamin biosynthesis I (*q*-trend < 0.001), and superpathway of (R,R)-butanediol biosynthesis (*q*-trend = 0.014; Fig. 2f; Supplementary Data 2) were depleted with higher fiber intake.

### Added sugar intake

**Fig. 3:**
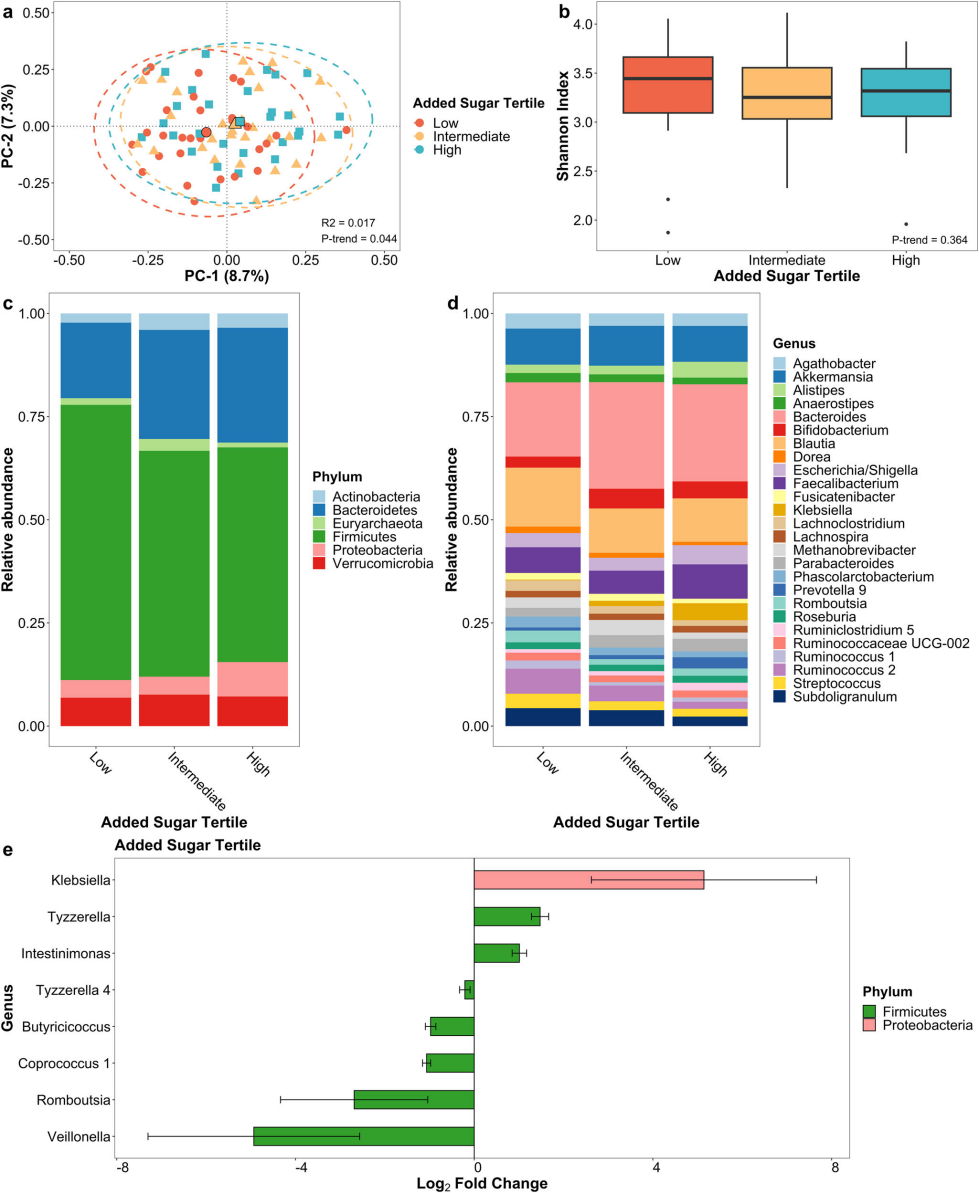
Gut microbiome of PD patients varies by added sugar intake. **a** Principal coordinate analysis plot of the microbiome by added sugar intake tertile. **b** Box plot of microbial diversity using Shannon index by added sugar intake tertile. Taxonomic summary plot of relative abundance at **c** phylum and **d** genus level (≥1% relative abundance) by added sugar intake tertile. **e** Differential abundance of microbial taxa at genus level by added sugar intake after adjusting for age, sex, race, and sequencing platform. Taxa in (**e**) are associated with categorical added sugar intake tertile at *q*-trend <0.05 and continuous added sugar intake at *q*-value < 0.05. Error bars represent 95% confidence intervals.

Added sugar intake was associated with beta diversity (*p*-trend = 0.044; Fig. 3a), but again not with alpha diversity (Fig. 3b). In contrast to the negative associations observed with higher HEI scores and fiber intake with principal coordinate axes, increasing tertiles of added sugar intake showed a positive association with the first and second principal coordinate axes. Furthermore, with increasing added sugar consumption, at the genus level, *Klebsiella* (*q*-trend = 0.001) was increased, while *Butyricicoccus* (*q*-trend < 0.001), *Coprococcus 1* (*q*-trend < 0.001) and *Romboutsia* (*q*-trend = 0.001; Fig. 3e; Supplementary Data 1) were decreased. There were no differences in the predicted metagenome linked with the added sugar intake (Supplementary Table 1).

### Sensitivity analysis

We stratified our analyses by constipation status, as PD patients with lower HEI score were more likely to be constipated, and constipation is known to influence the gut microbiome. Dietary fiber was associated with altered overall microbial composition in constipated patients (*p*-trend = 0.029), while added sugar intake showed a marginal association in both constipated and non-constipated patients (*p*-trend = 0.069 and *p*-trend = 0.058, respectively; Supplementary Table 2). Furthermore, both constipated and non-constipated patients with healthier diets had a higher abundance of the genus *Coprococcus 1* (*q*-trend <0.001 for both; *p*-heterogeneity = 0.771; Supplementary Data 3). However, in constipated patients, the effect of a healthy diet on *Coprococcus 1* was weaker than in non-constipated patients, suggesting a subtle yet consistent influence of diet on the microbiome within the constipated group.

We also stratified analyses by PD duration, as we reported previously that patients with longer disease duration consumed higher amounts of sugars^6^ and exhibited differences in microbial abundance with increasing disease duration^21^. Patients with both long (≥10 years) and short (<10 years) PD durations who maintained a healthier diet had an increased abundance of *Coprococcus 1* (*q*-trend <0.001 for both; *p*-heterogeneity = 0.160; Supplementary Data 4). A similar association was seen with higher fiber intake, which was associated with a greater abundance of *Coprococcus 1* in both duration groups (*q*-trend < 0.001 for both; *p*-heterogeneity = 0.016). Yet, for those with longer disease durations, the impact of a better diet on *Coprococcus 1* were less pronounced than for those with shorter durations, indicating diminished efficacy in the longer-duration group.

Furthermore, some microbes identified in the PD patients were similarly associated with diet in controls; specifically, *Ruminococcaceae UCG-003* increased with a better diet (*q*-trend < 0.001), while *Ruminococcaceae NK4A214 group* was more abundant with higher fiber intake (*q*-trend < 0.001; Supplementary Data 5 and 6). On the other hand, PD patients, but not controls, with greater added sugar intake had a higher abundance of *Klebsiella* (*q*-trend < 0.001; *p*-heterogeneity = 0.041; Supplementary Data 6). However, the gut microbial community of PD patients is quite distinct from controls, and we had limited statistical power to test for interactions.

## Discussion

Our data suggest that the gut microbial profiles of PD patients are influenced by dietary habits. We observed differences in the microbiome in relation to diet quality, as well as the intake of dietary fiber and added sugar. These findings align with previous studies and further suggest that a healthy diet may have beneficial effects on the gut microbiome in PD patients (Supplementary Table 3). Specifically, with a healthier diet, we observed a reduction in the abundance of putative pro-inflammatory bacteria that are generally found to be enriched in PD patients compared to controls. Furthermore, a healthy diet in our study participants increased the abundance of putative anti-inflammatory bacteria that are typically depleted in PD patients compared to controls. One implication of our findings is that the previously reported microbiome associations with PD may have been due, at least partially, to dietary differences between PD patients and control subjects.

In this study, we observed that a better diet (indicated by a higher HEI score and fiber intake) in PD patients was associated with an increase in SCFA-producing bacteria, including *Butyricicoccus*, *Coprococcus 1, Hydrogenoanaerobacterium*, *Negativibacillus*, *Romboutsia*, *Ruminococcaceae NK4A214 group*, and *Ruminococcaceae UCG-003*. These SCFA-producing bacteria, primarily known for producing butyrate, provide energy for intestinal epithelial cells, strengthen the intestinal epithelium, and reduce inflammation^22^. Moreover, the anti-inflammatory properties of SCFAs affect the enteric nervous system, promote normal microglia development, and potentially modulate inflammation in the central nervous system^23^.

On the flip side, a higher intake of added sugar in our study participants led to a decrease in *Butyricicoccus*, *Coprococcus 1*, and *Romboutsia*. In PD patients, a lower abundance of SCFA-producing bacteria has been found in a series of studies in comparison to healthy controls^9–13^. PD patients have higher levels of pro-inflammatory cytokines in the colon and serum, suggesting that they suffer from systemic inflammation, which could result in microglial activation that drives disease progression^24^. For instance, a lower abundance of *Butyricicoccus* was noted in patients with early PD and REM sleep behavior disorder^10^, and a lower abundance of *Romboutsia* was associated with worsening cognitive function and depressive symptoms in PD patients^25^.

We found that higher added sugar intake was associated with an increased in amyloid-producing bacteria, *Klebsiella*. In animal models, bacterial amyloids have been shown to increase alpha-synuclein production in the gut and its accumulation in the brain, thereby enhancing cerebral inflammation^26^. Additionally, we observed that a higher fiber intake was associated with a decrease in the pro-inflammatory bacteria, *Bacteroides*, which have been found to be elevated in PD patients compared to controls^12,27–29^. A higher abundance of *Bacteroides* was previously shown to be positively correlated with the severity of motor symptoms^29,30^. Overall, our analysis suggests that maintaining a healthy diet may benefit PD patients, with the potential of reducing both motor and non-motor symptoms and slowing disease progression.

Furthermore, in the early stages of PD, dietary habits may have a more pronounced effect on maintaining SCFA-producing bacteria, suggesting that in early PD, the microbiome may still be modifiable by diet. In contrast, in more advanced stages of PD, changes in the microbiome might be more entrenched and less responsive to dietary changes. The results of our analysis stratified by PD duration highlight the potential importance of early dietary interventions in PD and suggest that managing gut microbiota through diet could be more challenging as the disease progresses.

Our predicted functional pathway results further strengthen the taxa-based findings; a healthy diet reduces lipopolysaccharide biosynthesis and taurine degradation. In PD, lipopolysaccharide, an endotoxin from the outer membrane of Gram-negative bacteria, is implicated in neuroinflammation. Elevated circulating lipopolysaccharide, possibly entering the blood through a leaky gut barrier^31^, exacerbates neurodegeneration by activating microglia in the brain^32^. A previous study reported that lipopolysaccharide biosynthesis was increased in PD patients compared to health controls^33^. Healthy eating may potentially lower circulating lipopolysaccharide and mitigate systemic inflammation in PD. Our results also suggest that the reduction of taurine degradation in PD patients with higher HEI score may help maintain higher systemic levels of taurine, potentially providing neuroprotective effects on the nervous system with better diet quality. Recent studies have demonstrated that plasma taurine levels are sharply reduced in PD patients and inversely correlated with severity of motor symptoms, supporting the potential neuroprotective role of taurine in PD^34,35^. Furthermore, in a mouse model, increased taurine degradation was observed in PD mice with fewer *Ruminococcaceae*^36^. Our findings indicated that a better diet in PD patients was associated with higher levels of Ruminococcaceae family genera, which may support taurine metabolism and lead to reduced degradation of taurine by gut microbiota.

Our study reports several findings on gut microbial composition and diet in PD patients who were identified from the largest community-based PD study in the United States. However, limitations include a small sample size, which limits confounder control and the identification of less prevalent microbial taxa, and the cross-sectional design, which does not allow us to infer temporality or causality of associations. To address these limitations and further explore the impact of diet and the interaction between diet and the microbiome on the progression of PD, subsequent studies should leverage longitudinal data on diet, microbiome, and disease progression. In addition, the metagenomic pathways were based on predicted metagenomic data and not the actual metagenome. Therefore, future research should consider using shotgun sequencing and metabolomics to provide a more robust understanding of the impact of diet on gut microbiome composition and function in PD. Measurement errors inherent in DHQ II assessments may also have led to the misclassification of dietary exposures. In the future, we hope to be able to evaluate the stability of diet quality over time in our patient cohort, but for now we have to assume that the diet did not change in a major way during the gap between diet assessment and stool collection (average 6 months). Previous research established that the stability of HEI-2015 scores derived from the DHQ is stable over a 1-year period^37^, and that about 60% of gut microbial strains remain the same over a 5-year period^38^, providing support for the stability of both diet and gut microbiome over extended periods. We, however, acknowledge the need for additional data on the temporal stability of the diet and the microbiome in PD. Finally, our study population was primarily non-Hispanic white males, and while this is consistent with the prevalence of PD being 1.4 times higher in men than in women^39^, results may not be generalizable to populations with a different gender or racial/ethnic distribution.

In conclusion, we are linking the gut microbiome composition and function to the diet of PD patients. Overall diet quality, as well as fiber and added sugar intake, are associated with gut microbiome composition that was previously described in studies comparing PD patients to controls. A healthier diet increases the abundance of putative anti-inflammatory butyrate-producing bacteria while decreasing the abundance of putative pro-inflammatory bacteria in PD patients. Given these insights, consuming a high-quality diet may help address the pro-inflammatory dysbiosis in PD patients and possibly provide some protection against disease progression.

## Methods

### Study population

We conducted a cross-sectional analysis on a subgroup of PD patients who participated in the Parkinson’s Environment and Gene (PEG) study, a population-based case-control study in rural California that enrolled 832 PD patients at baseline. PD cases were recruited during 2001–2007 (PEG1) and 2011–2017 (PEG2). At baseline, eligible PD cases met the following criteria: (1) newly diagnosed within 3–5 years, (2) residing in California for at least five years, specifically in Fresno, Kern, or Tulare county at the baseline screening, (3) received a PD diagnosis from local providers, which was later confirmed by the University of California, Los Angeles (UCLA) movement disorder specialists, and (4) absence of other neurological conditions or a terminal illness. Patients who could be re-contacted between 2017–2020 (PEG-Gut) were requested to provide a fecal sample (*n* = 130).

For the following analyses, we further excluded patients with missing Diet History Questionnaire II (DHQ II) or gut bacteria 16S rRNA sequencing data, as well as those with implausible daily energy intakes (men: <500 or >5000 kcal/day, women: <400 or >4000 kcal/day). This resulted in a final cohort of 85 patients for the analyses. We collected information on demographics, medical histories, lifestyle, and work and home environments through standardized interviews. The workflow is in Supplementary Fig. 1. All patients provided written informed consent, and the study was approved by the Institutional Review Board of UCLA (IRB #21-000256 and IRB#11-001530).

### Diet and covariate assessment

All PD participants completed the DHQ II to assess their usual dietary intake over the past month prior to interview. On average, this assessment took place no more than half a year apart from the fecal sample collection date. Subsequently, we utilized the DHQ II data to compute the HEI-2015 as a measure of diet quality. This instrument reflects recommendations based on the 2015-2020 Dietary Guidelines of Americans and consists of 13 components with a total score ranging from 0 (worst) to 100 (best) points. The HEI score was expressed as a per interquartile range (IQR) increase.

We previously identified differences in dietary fiber and added sugar consumption between PD patients and household/community control participants^40^. In the current analyses, we further focused on these specific nutrients, expressed as a percentage of total energy intake. This nutrient density approach is suitable, as these nutrients contribute to dietary energy, allowing for comparison and standardization of nutrient intake^41^. Diet quality and nutrients were treated as continuous and categorical variables, categorized into tertiles of intake (HEI: ≤5.0, 5.0–5.8, >5.8 per 11.6-point increase; fiber: ≤0.9, 0.9–1.1, >1.1% energy; added sugar: ≤2.5, 2.5–3.8, >3.8% energy).

### Fecal sample collection and microbiome measurements

Participants were asked to collect fecal samples at their homes using a Para-Pak^®^ collection kit preserved in 95% ethanol. Samples were shipped to UCLA within 14 days of collection and stored at −80 °C in a freezer until analyses. Bacterial genomic DNA was extracted from fecal samples using the ZymoBIOMICS DNA kit, following the manufacturer’s protocol. As reported previously^42^, PCR amplification of the V4 region of the 16 rRNA gene was followed by 250 × 2 paired-end sequencing on Illumina HiSeq 2500 or MiSeq platforms. Sequence reads were quality-filtered using the DADA2 pipeline (v1.22.0). Quality-filtered reads were processed into amplicon sequence variants (ASVs) and assigned taxonomy based on the SILVA database^43^. ASVs were removed if the abundance was less than 50 or the prevalence was less than 20% in all samples (3,480,980/4,432,700 sequences remained after this filtering step). ASVs were also rarefied to even depth without abundance/prevalence filtering for assessing alpha diversity. The data processing steps described above were performed using the phyloseq packages (v1.34.0).

### Statistical analysis

Microbiome data were analyzed for alpha diversity, beta diversity, and the association of taxa abundance with diet quality and nutrients. Alpha diversity (within-subject diversity) was assessed using the Shannon diversity index, representing bacterial community evenness and richness. We examined whether diet features were associated with alpha diversity using linear regression models. Beta diversity (between-subject diversity) in relation to diet features was assessed using permutational multivariate analysis of variance (PERMANOVA) of the Bray-Curtis distance. Results of beta diversity were visualized using principal coordinate analysis. Differential abundance and the association of microbial genera with diet features were evaluated using negative binomial generalized linear models implemented in MaAsLin2 (v1.12.0)^44^. The minimum abundance was set to 0.01 and the minimum prevalence set to 0 as we already filtered our taxa previously. Additionally, as part of sensitivity analyses, we conducted stratified analyses by constipation status and PD duration (long: ≥10 years; short: <10 years) and re-assessed the microbiome-diet associations we observed in cases also in household and community controls.

The metagenomic profile of the gut microbiome was predicted based on 16S rRNA sequencing data. Functional inferences of MetaCyc pathway abundances were made using the PICRUSt2 (v2.4.1) algorithm^45^. Metacyc pathways were removed if the abundance was less than 100 or the prevalence was less than 20% in all samples (340/400 pathways remained after filtering). We also assessed differences in alpha and beta diversity and the abundance of Metacyc pathways by diet.

All regression models were adjusted for age, sex, race/ethnicity (non-Hispanic white, non-white or Hispanic), and sequencing platform (HiSeq, MiSeq), with diet as categorical or continuous exposure variables. For trend tests, we used the midpoint value of each diet tertile as a continuous variable to minimize the influence of outliers. Additional factors we initially explored but did not include in the final model include the use of PD medication (levodopa or dopamine agonist dosage), vitamin intake, and supplement intake. These factors did not change reported results more than minimally and this approach avoids sparse data issues. All statistical tests were two-sided. Taxa and pathways we considered differential in abundance were associated with diet at a *q*-trend <0.05 for categorical variables and a *q*-value < 0.05 for continuous variables. The analyses were performed using R (v4.1.2).

### Reporting summary

Further information on research design is available in the Nature Research Reporting Summary linked to this article.

### Supplementary information

Supplementary Material

Reporting Summary

Supplementary Data

## Data Availability

The data generated and analyzed during the current study are available to the public at the NCBI Sequence Read Archive under BioProject ID PRJNA1101026. Further data are available upon request from the corresponding author, B.R.

## Code availability

All software, packages, their versions, relevant specifications, and parameters are stated in the Methods section. No custom codes are used. The code for performing high-resolution sequencing filtering, statistical analyses, and figure generation is provided at https://github.com/dayoonkwon/PEGDiet.
