# Supplementary material for: Diet and the gut microbiome in patients with Parkinson’s disease
Source: NPJ Parkinsons Dis. 2024 Apr 22;10:89. doi: 10.1038/s41531-024-00681-7 (PMC11035608; doi:10.1038/s41531-024-00681-7)
Supplement: Supplementary file 1 — Supplementary Material [file 41531_2024_681_MOESM1_ESM.pdf]

SUPPLEMENTARY MATERIAL

Supplementary Figure 1. The STORMS flowchart. Following reporting guidelines for human microbiome research, we show the step-by-step process by which (a) the study was conducted, enrollment and data collection for PD patients, (b) sequencing and bioinformatics pipeline, and (c) statistical analyses.

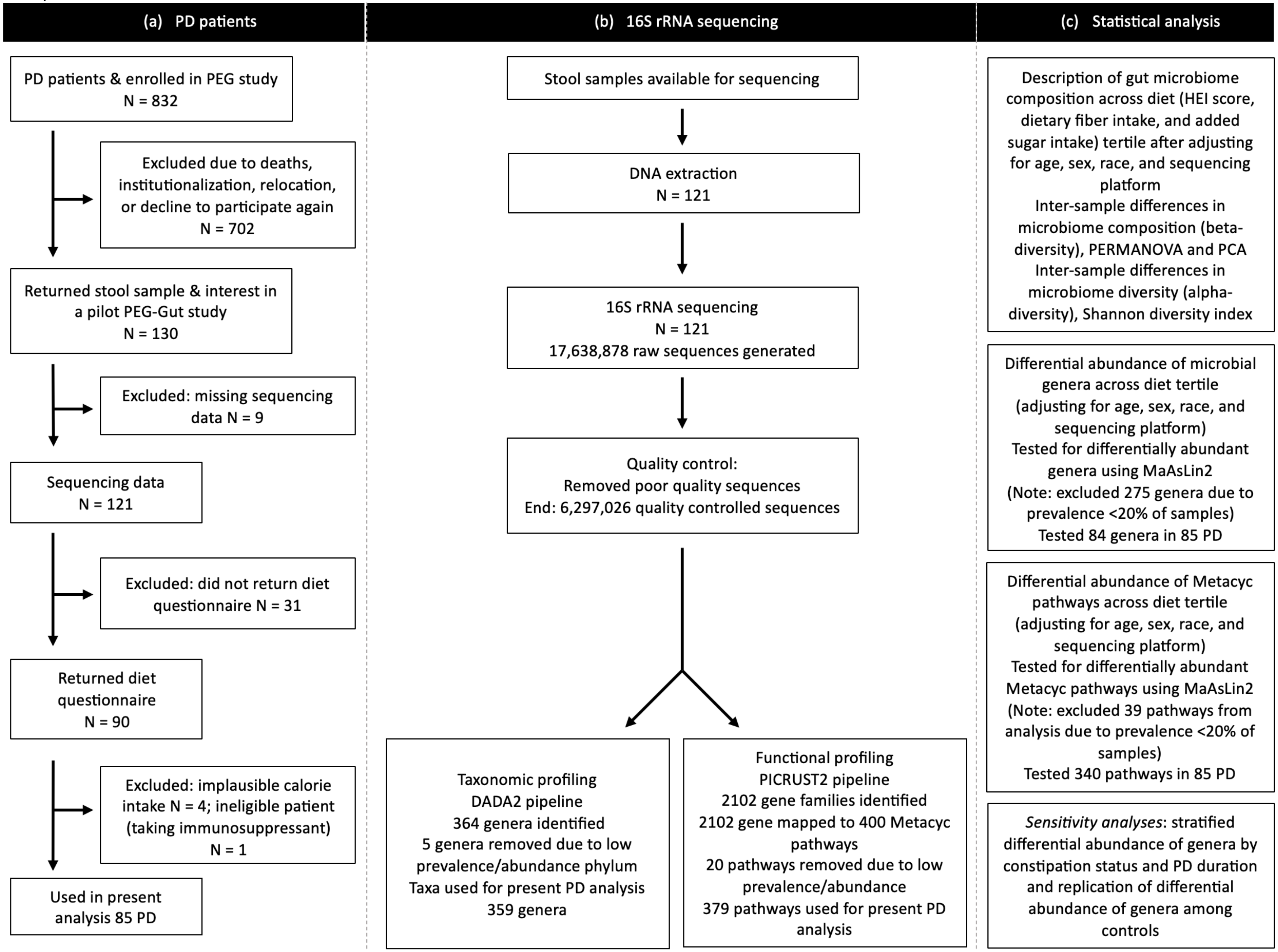

Supplementary Table 1. Association of beta diversity of predicted bacterial functional genes with diet. Parameters are from Bray-Curtis distance with continuous diet as the predictor, adjusting for age, sex, race, and platform. Trend tests across tertile are done by entering the tertile midpoints into the model as continuous term.

Bray-Curtis distance	Categorical tertile		Continuous	
	**R2**	**p-trend**	**R2**	**p-value**
HEI	0.03182	0.0298	0.02272	0.0744
Dietary fiber	0.02074	0.0912	0.02599	0.0497
Added sugar	0.01589	0.1598	0.01425	0.1981

Supplementary Table 2. Association of gut microbial beta diversity with diet stratified by constipation. Parameters are from Bray-Curtis distance with continuous diet as the predictor, adjusting for age, sex, race, and platform. Trend tests across tertile are done by entering the tertile midpoints into the model as continuous term.

	**Constipation (n = 50)**				**No constipation (n = 35)**			
	**Categorical tertile**		**Continuous**		**Categorical tertile**		**Continuous**	
**Bray-Curtis distance**	**R2**	**p-trend**	**R2**	**p-value**	**R2**	**p-trend**	**R2**	**p-value**
HEI	0.02357	0.1898	0.02341	0.2043	0.03361	0.2098	0.03804	0.0870
Dietary fiber	0.02927	0.0289	0.02820	0.0395	0.02150	0.9193	0.03598	0.1259
Added sugar	0.02676	0.0694	0.02584	0.0956	0.03956	0.0575	0.02143	0.9218

Supplementary Table 3. Table presenting results of changes of gut microbiota compositions associated with Parkinson's disease and diet. ↑ and color coded blue indicate higher abundance associated with increased diet or PD. ↓ and color coded orange indicate lower abundance associated with increased diet or PD. Empty cells indicate that no significant changes. Putative anti-inflammatory bacteria are marked in green. Putative pro-inflammatory bacteria are marked in yellow. PD = Parkinson's disease, HEI = healthy eating index.

	**Changes associated with PD (previously reported in literature)**		**Changes with diet in PD patients (observed in this study)**		
**Genus**	**PD vs. healthy controls**	**Associated clinical characteristics**	**HEI**	**Fiber**	**Added sugar**
Adlercreutzia				↑	
Butyricicoccus	↓[1,2]	↓Patients with RBD and early PD[2]	↑	↑	↓
Coprococcus 1	↓[3-5]		↑	↑	↓
Hydrogenoanaerobacterium		↓Patients with lower MoCA scores[6]		↑	
Negativibacillus			↑	↑	
Romboutsia		↓ PD patients with worsening cognitive function and depressive symptoms[7]			↓
Ruminococcaceae NK4A214 group	↓[8-10]	↓PD patients with tremor dominant phenotype[9]; use of COMT inhibitors[10]		↑	
Ruminococcaceae UCG-003	↓[8-10]	↓PD patients with tremor dominant phenotype[9]; use of COMT inhibitors[10]	↑		
Subdoligranulum			↑		
Turicibacter	↑[11]		↑		
Tyzzerella 4		↓ Patients with cognitive impairment[12]	↑	↑	↓
Veillonella	↑[13,14]				↓
Bacteroides	↑[5,8,15,16]	↑PD patients with tremors[13], postural instability and gait disability phenotypes[16], and severe motor symptoms[13]		↓	
Eggerthella	↑[17]		↓		
Faecalitalea			↓		
Klebsiella	↑[15,18]				↑
Lachnospiraceae NK4A136 group	↓[2,5,14,18-22]	↓Patients with RBD and early PD[2]	↓		
Parabacteroides	↑[13,20]		↓	↓	
Tyzzerella			↓	↓	↑
UC5-1-2E3 (Lachnospiraceae family genus)			↓	↓	
Butyricimonas	↑[13]		↓	↑	

Intestinimonas	↑[19]				↓	↑	↑

REFERENCES

1 Lubomski, M. *et al.* The impact of device-assisted therapies on the gut microbiome in Parkinson's disease. *J Neurol* **269**, 780-795 (2022). https://doi.org/10.1007/s00415-021-10657-9

2 Huang, B. *et al.* Gut microbiome dysbiosis across early Parkinson's disease, REM sleep behavior disorder and their first-degree relatives. *Nature Communications* **14** (2023). https://doi.org/ARTN 2501 10.1038/s41467-023-38248-4

3 Chen, Y., Xu, J. & Chen, Y. Regulation of Neurotransmitters by the Gut Microbiota and Effects on Cognition in Neurological Disorders. *Nutrients* **13** (2021). https://doi.org/10.3390/nu13062099

4 O'Donovan, S. M. *et al.* Nigral overexpression of alpha-synuclein in a rat Parkinson's disease model indicates alterations in the enteric nervous system and the gut microbiome. *Neurogastroenterol Motil* **32**, e13726 (2020). https://doi.org/10.1111/nmo.13726

5 Keshavarzian, A. *et al.* Colonic bacterial composition in Parkinson's disease. *Mov Disord* **30**, 1351-1360 (2015). https://doi.org/10.1002/mds.26307

6 Ren, T. *et al.* Gut Microbiota Altered in Mild Cognitive Impairment Compared With Normal Cognition in Sporadic Parkinson's Disease. *Front Neurol* **11**, 137 (2020). https://doi.org/10.3389/fneur.2020.00137

7 Xie, A. J. *et al.* Bacterial Butyrate in Parkinson's Disease Is Linked to Epigenetic Changes and Depressive Symptoms. *Movement Disord* **37**, 1644-1653 (2022). https://doi.org/10.1002/mds.29128

8 Gerhardt, S. & Mohajeri, M. H. Changes of Colonic Bacterial Composition in Parkinson's Disease and Other Neurodegenerative Diseases. *Nutrients* **10** (2018). https://doi.org/10.3390/nu10060708

9 Weis, S. *et al.* Effect of Parkinson's disease and related medications on the composition of the fecal bacterial microbiota. *NPJ Parkinsons Dis* **5**, 28 (2019). https://doi.org/10.1038/s41531-019-0100-x

10 Barichella, M. *et al.* Unraveling gut microbiota in Parkinson's disease and atypical parkinsonism. *Movement Disord* **34**, 396-405 (2019). https://doi.org/10.1002/mds.27581

11 Baldini, F. *et al.* Parkinson's disease-associated alterations of the gut microbiome predict disease-relevant changes in metabolic functions. *BMC Biol* **18**, 62 (2020). https://doi.org/10.1186/s12915-020-00775-7

12 Lu, S. *et al.* Gut Microbiota and Targeted Biomarkers Analysis in Patients With Cognitive Impairment. *Front Neurol* **13**, 834403 (2022). https://doi.org/10.3389/fneur.2022.834403

13 Lin, C. H. *et al.* Altered gut microbiota and inflammatory cytokine responses in patients with Parkinson's disease. *J Neuroinflammation* **16**, 129 (2019). https://doi.org/10.1186/s12974-019-1528-y

14 Vascellari, S. *et al.* Gut Microbiota and Metabolome Alterations Associated with Parkinson's Disease. *mSystems* **5** (2020). https://doi.org/10.1128/mSystems.00561-20

15 Wallen, Z. D. *et al.* Metagenomics of Parkinson's disease implicates the gut microbiome in multiple disease mechanisms. *Nat Commun* **13**, 6958 (2022). https://doi.org/10.1038/s41467-022-34667-x

16 Mehanna, M. *et al.* Study of the gut microbiome in Egyptian patients with Parkinson's Disease. *BMC Microbiol* **23**, 196 (2023). https://doi.org/10.1186/s12866-023-02933-7

17 Zhang, K. *et al.* Parkinson's Disease and the Gut Microbiome in Rural California. *J Parkinsons Dis* **12**, 2441-2452 (2022). https://doi.org/10.3233/JPD-223500

18 Pietrucci, D. *et al.* Dysbiosis of gut microbiota in a selected population of Parkinson's patients. *Parkinsonism Relat Disord* **65**, 124-130 (2019). https://doi.org/10.1016/j.parkreldis.2019.06.003

19 Aho, V. T. E. *et al.* Gut microbiota in Parkinson's disease: Temporal stability and relations to disease progression. *EBioMedicine* **44**, 691-707 (2019). https://doi.org/10.1016/j.ebiom.2019.05.064

20 Barichella, M. *et al.* Unraveling gut microbiota in Parkinson's disease and atypical parkinsonism. *Mov Disord* **34**, 396-405 (2019). https://doi.org/10.1002/mds.27581

21 Hill-Burns, E. M. *et al.* Parkinson's Disease and Parkinson's Disease Medications Have Distinct Signatures of the Gut Microbiome. *Movement Disord* **32**, 739-749 (2017). https://doi.org/10.1002/mds.26942

22 Cirstea, M. S. *et al.* Microbiota Composition and Metabolism Are Associated With Gut Function in Parkinson's Disease. *Mov Disord* **35**, 1208-1217 (2020). https://doi.org/10.1002/mds.28052
