# Supplementary material for: Diet and the gut microbiome in patients with Parkinson’s disease
Source: NPJ Parkinsons Dis. 2024 Apr 22;10:89. doi: 10.1038/s41531-024-00681-7 (PMC11035608; doi:10.1038/s41531-024-00681-7)
Supplement: Supplementary file 2 — Reporting Summary [file 41531_2024_681_MOESM2_ESM.pdf]

nature portfolio

Corresponding author(s): Beate Ritz

Last updated by author(s): Oct 11, 2023

Reporting Summary

Nature Portfolio wishes to improve the reproducibility of the work that we publish. This form provides structure for consistency and transparency in reporting. For further information on Nature Portfolio policies, see our Editorial Policies and the Editorial Policy Checklist.

Statistics

For all statistical analyses, confirm that the following items are present in the figure legend, table legend, main text, or Methods section.

n/a	Confirmed	
☐	☒	The exact sample size (*n*) for each experimental group/condition, given as a discrete number and unit of measurement
☐	☒	A statement on whether measurements were taken from distinct samples or whether the same sample was measured repeatedly
☐	☒	The statistical test(s) used AND whether they are one- or two-sided<br>*Only common tests should be described solely by name; describe more complex techniques in the Methods section.*
☐	☒	A description of all covariates tested
☐	☒	A description of any assumptions or corrections, such as tests of normality and adjustment for multiple comparisons
☐	☒	A full description of the statistical parameters including central tendency (e.g. means) or other basic estimates (e.g. regression coefficient) AND variation (e.g. standard deviation) or associated estimates of uncertainty (e.g. confidence intervals)
☐	☒	For null hypothesis testing, the test statistic (e.g. *F*, *t*, *r*) with confidence intervals, effect sizes, degrees of freedom and *P* value noted<br>*Give P values as exact values whenever suitable.*
☒	☐	For Bayesian analysis, information on the choice of priors and Markov chain Monte Carlo settings
☐	☒	For hierarchical and complex designs, identification of the appropriate level for tests and full reporting of outcomes
☐	☒	Estimates of effect sizes (e.g. Cohen's *d*, Pearson's *r*), indicating how they were calculated

Our web collection on statistics for biologists contains articles on many of the points above.

Software and code

Policy information about availability of computer code

Data collection	No software was used.
Data analysis	R (v4.1.2); DADA2 (v1.22.0); phyloseq (v1.34.0); ggplot2 (v3.4.2); MaAsLin2 (v1.12.0); PICRUSt2 (v2.4.1)

For manuscripts utilizing custom algorithms or software that are central to the research but not yet described in published literature, software must be made available to editors and reviewers. We strongly encourage code deposition in a community repository (e.g. GitHub). See the Nature Portfolio guidelines for submitting code & software for further information.

Data

Policy information about availability of data

All manuscripts must include a data availability statement. This statement should provide the following information, where applicable:

- Accession codes, unique identifiers, or web links for publicly available datasets
- A description of any restrictions on data availability
- For clinical datasets or third party data, please ensure that the statement adheres to our policy

All data are available from the corresponding author upon reasonable request.

Research involving human participants, their data, or biological material

Policy information about studies with human participants or human data. See also policy information about sex, gender (identity/presentation), and sexual orientation and race, ethnicity and racism.

Reporting on sex and gender	We considered gender in this manuscript. This information was self-reported by participants. In total, our study analyzed data from 57 men and 28 women (shown in Table 1). Parkinson's disease is more common among men, therefore our statistical analysis controlled for gender as a covariate.
Reporting on race, ethnicity, or other socially relevant groupings	We considered race/ethnicity in this manuscript. This information was self-reported by participants. In total, our study analyzed data from 69 White non-Hispanic and 16 non-While or Hispanic patients (shown in Table 1). Our statistical analysis also controlled for race/ethnicity as a covariate.
Population characteristics	This manuscript was based on 85 Parkinson's patients. The mean age of the patients was 62 (SD = 8.6). 81% of the patients were of European ancestry; 67% of the patients were male. This information along with more detailed population characteristics is provided in Table 1.
Recruitment	Study enrollment for the epidemiologic study took place over two waves: wave 1 (PEG1): 2001-2007 (n = 357 PD patients) and wave 2 (PEG2): 2011-2017 (n = 472 PD patients). Patients were first (PEG1) identified through large medical groups, neurologists, and public service announcements and second (PEG2) from the pilot PD registry program in California. Patients who could be re-contacted between 2017-2020 (PEG-Gut) were requested to provide a fecal sample (n = 130). A detailed description of enrollment is provided in the methods.
Ethics oversight	The PEG study was approved by the UCLA Institutional Review Board (IRB#21-000256 and IRB#11-001530) and informed consent was obtained from all individuals. Our research conformed to the Declaration of Helsinki.

Note that full information on the approval of the study protocol must also be provided in the manuscript.

Field-specific reporting

Please select the one below that is the best fit for your research. If you are not sure, read the appropriate sections before making your selection.

☒ Life sciences ☐ Behavioural & social sciences ☐ Ecological, evolutionary & environmental sciences

For a reference copy of the document with all sections, see nature.com/documents/nr-reporting-summary-flat.pdf

Life sciences study design

All studies must disclose on these points even when the disclosure is negative.

Sample size	The sample size was based on available data from the PEG-Gut study (n = 85).
Data exclusions	We excluded patients with missing Diet History Questionnaire II or 16S rRNA sequencing data, and those with implausible daily energy intakes (<500 or >5,000 kcal/day for men and <400 or >4,000 kcal/day for women).
Replication	We used two study waves from the PEG population. These study waves are independent populations, recruited from the same geographic area, with the same study protocol (neurologic exam, patient questionnaire, exposure assessment, etc). This allowed us to assess replication from one study wave to the other.
Randomization	We conducted analysis of existing data from an observational study. The study subjects were not randomly assigned to exposure groups, but compared based on diet quality (Healthy Eating Index-2015), fiber intake, and added sugar intake.
Blinding	We conducted analysis of existing data from an observational study. Thus blinding was not relevant.

Reporting for specific materials, systems and methods

We require information from authors about some types of materials, experimental systems and methods used in many studies. Here, indicate whether each material, system or method listed is relevant to your study. If you are not sure if a list item applies to your research, read the appropriate section before selecting a response.

Materials & experimental systems

n/a	Involved in the study
☒	☐ Antibodies
☒	☐ Eukaryotic cell lines
☒	☐ Palaeontology and archaeology
☒	☐ Animals and other organisms
☒	☐ Clinical data
☒	☐ Dual use research of concern
☒	☐ Plants

Methods

n/a	Involved in the study
☒	☐ ChIP-seq
☒	☐ Flow cytometry
☒	☐ MRI-based neuroimaging

Plants

Seed stocks	*Report on the source of all seed stocks or other plant material used. If applicable, state the seed stock centre and catalogue number. If plant specimens were collected from the field, describe the collection location, date and sampling procedures.*
Novel plant genotypes	*Describe the methods by which all novel plant genotypes were produced. This includes those generated by transgenic approaches, gene editing, chemical/radiation-based mutagenesis and hybridization. For transgenic lines, describe the transformation method, the number of independent lines analyzed and the generation upon which experiments were performed. For gene-edited lines, describe the editor used, the endogenous sequence targeted for editing, the targeting guide RNA sequence (if applicable) and how the editor was applied.*
Authentication	*Describe any authentication procedures for each seed stock used or novel genotype generated. Describe any experiments used to assess the effect of a mutation and, where applicable, how potential secondary effects (e.g. second site T-DNA insertions, mosiacism, off-target gene editing) were examined.*
